# Book Reviews

**Published:** 1909-01

**Authors:** 


### BOOK REVIEWS

GENERAL SURGERY. A presentation of the scientific principles upon which the practice of modern surgery is based, by Ehrich Lexer, M. D., Professor of Surgery, University of Konegsberg. American edition edited by Arthur Dean Bevan, M. D., Professor and Head of the Department of Surgery, Rush Meical College in Affiliation with the University of Chicago. Authorized translation of the second German edition by Dean Lewis, M. D., Assistant Professor of Surgery, Rush Medical College, with 449 illustrations. D. Appleton & Co., New York.

This excellent volume on the principles seems to have lost none of its original value by its translation, but if anything has gained on account of the able editorship by Bevan and the careful translation by Lewis. All teachers we think admit that general or the science and art of surgery should be taken up before the student studies special or regional surgery. This book affords the student almost an ideal work from which to acquire such knowledge. If there i sany criticism to make of the above book for such purposes it is that it is rather more comprehensive than the limited time of the student permits. Its greatest value will result to practitioners who are interested in surgery; they may read this book with great interest and profit. The busy surgeon will also find in its pages, clearly stated, all of the recent advances in the science of surgery, as for example the significance and importance of the modern conception of infection and immunity, and the application of this knowledge to surgery. Lexer has presented his views in a clear, concise and practical manner. Dr. Oliver Ormsby has written a comprehensive chapter on blastomycosis. We feel safe in recommending this work as the most valuable and comprehensive presentation of the scientific principles upon which surgery is based.

PROGRESSIVE MEDICINE, VOL. IV, DECEMBER, 1908.

A Quarterly Digest of Advances, Discoveries and Improvements in the Medical and Surgical Sciences. Edited by Hobart Amory Hare, M. D., Professor of Therapeutics and Materia Medica in the Jefferson Medical College of Philadelphia. Octavo, 333 pages, with 26 engravings and 2 colored plates. Per annum, in four paper-bound volumes, containing over 1,200 pages, $6.00, net; in cloth, $9.00, net. Lea & Febiger, Publishers, Philadelphia and New York.

The December issue of *Progressive Medicine* is fully abreast of the reputation of this quarterly for practical usefulness to every active medical man, whether physician, surgeon or specialist. In fact, its contents are purposely limited to the clinical as distinguished from the theoretical aspects of medicine. As brief examples of these characteristics, we may cite only a few of the multitude of topics treated by Dr. Edsall, of Philadelphia, in his 80 pages on Diseases of the Digestive System, if possible the most important in the entire range of human ailment. He points out the clinical bearings of recent physiological researches on the stomach and of psychic influences on digestion, deals with the results of recent x-ray advances in connection with that organ, devotes 10 pages to Gastric Ulcers, Stenosis and Carcinoma, revises to date the recently developed subject of intestinal diverticula, and illuminates the hitherto obscure field of diseases of the pancreas. In the same most cursory manner we may refer to the articles on Renal Tuberculosis and Syphilitic Nephritis in the secretion on the Kidneys, written by Dr. John Rose Bradford, of London. Bloodgood, of Baltimore, has covered in a hundred pages, the real additions to practical surgery during the year. His remarks on Surgical Shock deal instructively with a common and serious condition. He devotes twenty-five pages to advances in Surgery of the Blood-vessels, a subject of especial interest at the present time, and the same may be said of his articles on Surgery of the Joints. He closes with twenty pages on Tumors, thus completing in connection with his successive sections on these morbid growths, the most important monograph on the subject in the language. Belfield, of Chicago, covers the latest advances in the Genito-Urinary field authoritatively in thirty pages. The Assistant Editor, Dr. Landis, closes the year with a Practical Therapeutic Referendum, reviewing the advances in both medical and non-medical treatment, and giving due prominence to untoward results following serum therapy.

---

GONORRHOEA IN WOMEN. By Palmer Findley, M. D., Prof. of Gynecology in the College of Medicine of the University of Nebraska, Omaha, etc. Published by C. V. Mosby Medical Book and Publishing Co., St. Louis, Mo.

In the above work Findley has collected much of value from the literature bearing upon gonorrhoea in women and especially as regards the sociologic consideration of this important subject. While the author does not deal with question as fully as one might expect in a monograph upon the subject, he has collected much of importance and such books, no doubt, will do much to open the eyes of the profession to a better understanding of the possible and likely sequels of uncured gonorrhoea in a careless husband, even though his disease is slight. While Findley has presented much of value, the matter cannot be said to be well digested; the style is careless and there is considerable repetition of matter in the quotations. The author speaks of the *inoculation* of gonorrhoea being the time elapsing between intercourse and the onset of the symptoms; we believe incubation should be used instead of inoculation.

Instead of digesting the matter to be presented and then giving it in his own language, he has quoted verbation quite freely from numerous authors; the disconnection thus produced makes the work seem more like a series of valuable abstracts than a book.

In conclusion we would say that our criticism is rather of the method of presenting the matter than as to its truth or importance and we would consequently recommend that all who have vague ideas as to the frequency and danger of gonorrhoea and as to its scientific management in women, obtain Findley's book and read it.

---

GENITO-URINARY DISEASES AND SYPHILIS. By Edgar G. Ballenger, M. D., Lecurer on Genito-Urinary Diseases, Syphilis and Urinalysis Atlanta School of Medicine, etc. With 85 illustrations, 276 pages. Atlanta, Ga.; E. W. Allen & Co., Publishers. (Price, $3.00.)

Wonder is frequently expressed that the medical profession of the South does not write more books. Southern doctors have the talent and the opportunity to produce as valuable a medical liturature as those of any section of the country, and the indications are that the younger generation is awakening to this fact, and will give the world more abundantly in book form the results of its study and experience.

It is an event of unusual interest when a Southern physician does venture into such authorship. This is especially true when one succeeds as admirably as has Dr. E. G. Ballenger, of Atlanta, in his "Genito-Urinary Diseases and Syphilis," which has just come from the press.

For the place this book is intended to fill, it seems to be ideal. It is not put forth as an exhaustice treatise on the subject named, and yet it is far from being a compend. It includes all that is essential and up-to-date, and omits historical references and obsolete methods and illustrations.

The student and general practitioner will find in the book everything that they will ask to know in the management of ordinary cases of genito-urinary diseases and syphilis, and will be able to find it without having to digest unnecessary detail. The matter is well arranged, and handled clearly and accurately.

D. Ballenger is to be congratulated upon the pains he has taken with the volume, and upon the happy result he has achieved.

Frank K. Boland, A. B., M. D.

### THE BORDERLAND OF DISEASE.

There is a growing tendency on the part of medical men to recognize the pathological importance of certain, at present, little understood conditions of the blood. Some of these indeterminable deviations from the normal present none of the aspects of the anemias, but nevertheless bear a direct relation to increased susceptability to bacterial infection. The studies of Wright on the opsonins, so called, are of special interest in this direction, inasmuch as they have in a measure converted many of our abstract theories into concrete facts. That certain constituents of the blood may be diminished without apparent decrease of the corpuscular elements or of the hemoglobin, is at last fairly well established, and while the specific properties of these constituents are not as yet definitely known, there is abundant reason for attributing certain phases of malnutrition, as well as a general lowering of organic resistance to bacteria, to their absence or decrease. The clinical expression of this blood weakness, or chemico-physiologic deficiency, is subject to great variation, but the symptom-complex usually consists of a general physical decline, loss of weight, increased tendency to fatigue, and a fickle or decreased appetite,—all of which go to make up a picture of what is usually loosely termed general debility. In addition, when the blood dyscrasia is marked, two objective symptoms are frequently noted. These are slight transitory enlargement of the cervical lymphatics, and a marked susceptibility of the skin to abrasions and infection. imple injuries produce wounds that heal poorly and the processes of repair seem to be very feeble and inadequate.

This then in a general way constitutes what may be called the borderland of disease, a condition which even if it does not always preced tuberculosis, typhoid fever, pneumonia and many other diseases, certainly favors their development and tends to increase their severity.

The correction of this indefinite but none the less dangerous state of the blood is always urgent, particularly because of the favorable opportunities presented for increasing the resistance to those diseases to which it predisposes.

Regulation of the diet, careful attention to the personal hygiene, and as much outdoor living as possible are the essential features of the careful treatment of this condition of blood depravity. A good tonic is quite necessary in connection with the foregoing, and Pepto-Mangan (Gude) has been found very effective. Its pronounced hematogenic action is well-known, and the rapid hematosis which result from its administration unquestionably has a decided influence in coincidently raising the relatve immunizing power of the blood. Reparative processes in wounds are stimulated, simple glandulous swellings disappear, and tangible improvement in the general bodily nutrition rapidly follows. All this is accomplished, moreover, without placing the slightest tax on the digestive tract, and the patient is thus able not only to derive the fullest benefits from every effort in his behalf, but the course of his recovery is progressive and unbroken. His vital resistance is materially raised and the balance of functional vigor restored to the normal. That the extent to which this is accomplished measures the decreased liability to infectious disease, can no longer be doubted.

### THE NEUTRALIZATION OF DYSCRASIA.

In a very excellent article on "Various Forms of Headache" which appeared in *"Medical Progress"* a short time ago, Dr. J. U. Ray, of Blockton, Ala., states that "we must not only be particular to give a remedy intended to counteract the cause which produces headache, but we must also give an anodyne which will relieve the pain until the constitutional dyscrasia to which this trouble is due, has been neutralized. To answer this purpose, two antikamnia tablets will be found a safe and convenient reemdy. Usually they relieve the pain within twenty minutes. When we have a patient subject to sick headaches, we should caution him to keep his bowels regular, and when he feels the first premonition of an attack, he should take two antikamnia tablets. Most all patients tell us they know by certain symptoms when an attack is about to come. To these patients we can do nothing better than give them antikamnia tablets to be carried around with them always ready for use. They are prompt in action, and can be depended upon to produce the most soothing anodyne action. In this country and also in England these tablets are largely employed, with results that have caused them to be depended upon by the best observers in both countries. The remedy, having none of the drawbacks common to other agents of this class, it is eminently fitted to be applied in the treatment of the cases just described."

---

### SAN FRANCISCO BRANCH HOUSE OF THE H. K. MULFORD COMPANY.

The above engraving illustrates the San Francisco Branch House established by the H. K. Mulford Company at Second and Natoma streets, to provide better service to the jobbing and retail druggists of the Pacific Coast.

The high standard of Mulford's Biological Products, embracing Antitoxin, Curative Sera, Vaccines and Bacterins, is unquestioned. The firm is making special strides in the pharmaceutical business, and attention is directed to the fact that their pharmaceutical laboratories in Philadelphia and their biological laboratores in Glenolden are always open to representatives of both the medical and the pharmaceutical professions.

They are leaders in chemically-assayed and physiologically standarized drugs.

